# The Hospital. Nursing Section

**Published:** 1902-12-06

**Authors:** 


// The Hospital.

**Nursing Section.**

Contributions for this Section of "The Hospital" should be addressed to the Editor, "The Hospital" Nursing Section, 28 & 29 Southampton Street, Strand, London, W.C.

No. 845.—Vol. XXXIII. *SATURDAY, DECEMBER 6, 1902.*

### Notes on News from the Nursing World.

### THE LORDS COMMISSIONERS OF THE ADMIRALTY AS A NURSING AUTHORITY.

We print in another column the new regulations for Queen Alexandra's Royal Naval Nursing Service because it is possible that they may be useful to some of our readers for reference. But otherwise they are of very little consequence. Last May the head sister at Haslar Hospital informed our Commissioner that important changes were pending, but the authorities appear to be practically satisfied with the state of affairs which existed a year ago. The salary of the nursing sisters is henceforth to commence at £37 10s., instead of £30, rising by annual increments as hitherto to £50, and the head sisters at Plymouth and Chatham are now to receive the same remuneration as the head sister at Haslar. But, although it is notorious that the existing staff is not large enough to permit of the members taking the time off duty which is allowed them by regulations, no increase is made in the number of sisters. The ward master is still to be maintained, and the constantly changing male attendants will continue to be attached to the wards as nurses. Instead of the regulations being, in any sense, framed on the lines of those of Queen Alexandra's Military Nursing Service, which, whatever criticism they have provoked, are permeated by the spirit of reform, those of the Naval Nursing Service present a remarkable contrast. No attempt has, in fact, been made to render the latter worthy of the honoured and illustrious name it bears. Queen Alexandra is, indeed, the head; the new badge, which has already been described in our columns, is adorned by her monogram, and she signs the commissions. But the Lords Commissioners of the Admiralty have not thought it worth while to admit any qualified women to their councils, they have not appointed matrons to the service, and altogether they have shown by a policy of inaction how completely they are unable to understand the requirements of the times.

### TO NURSES OF THE PENSION FUND.

The honorary secretary of the Junius S. Morgan Benevolent Fund asks us to remind nurses that if they wish that their contributions to the benevolent fund should be included in the list for this year they should send them immediately to the Secretary of the Pension Fund, 28 Finsbury Pavement, E.C. The hon. secretary feels that nurses will be sorry to learn that although the number of members of the pension fund is continuously larger, fewer contribute towards their benevolent fund. In 1900 the nurses contributed £64; up to the present date only £29 has been received this year. The pleasant feature in the case is that the subscriptions include many from nurses who have benefited through the benevolent fund, to which they express themselves most grateful.

### SHAM CO-OPERATION AT GLASGOW.

The members of the Glasgow and West of Scotland Co-operation of Trained Nurses are to be congratulated upon the defeat of an attempt to impose upon them an entirely new constitution without anything like adequate consideration. We trust, moreover, that the amendment which was carried at the annual meeting by the votes of the nurses present, postponing the adoption of the scheme approved by the executive presages the absolute abandonment of it. As Mr. Bannatyne, who has taken the lead against it, pointed out at the annual meeting, the fact that the changes recommended by the executive would put the nurses who belong to the Co-operation in the position of servants, liable to be dismissed at a moment's notice, although they contribute 7½ per cent. out of their earnings to the up-keep of the home, is a fatal flaw. The report read at the meeting shows that the organisation is in a flourishing condition, and the Lord Provost, who was in the chair, was justified in the satisfaction he expressed at its success. But why not, then, let well alone? At any rate, it is for the nearly two hundred members attached to the Co-operation, and not for the executive, to determine whether they will surrender any of the rights which they possess under existing circumstances for the sake of advantages that may prove illusory.

### THE PROPOSED CHANGES IN WORKHOUSE NURSING.

The report of the Departmental Committee on the nursing of the sick poor in workhouses, together with the minutes of evidence, which were sent to us on Tuesday, occupy 250 pages, and will doubtless receive the serious consideration of all persons interested in the subject. In addition to the recommendations we were able to cite last week, the committee advise that the existing number of probationers be increased so as to allow for the defection of nurses who, having been trained at the infirmaries, do not afterwards continue in the service of the Local Government Board. The Committee do not believe that there will be any difficulty in securing sufficient candidates for probationerships, more especially in view of the establishment of the proposed four grades. Of course, a "trained nurse" would continue to be a nurse who has received three years' training at a major training school, with a resident medical officer, but the "qualified nurses," whom the Committee advise should be created, would be trained for at least one year at a minor training school and receive a certificate. The essentials of a minor training school are, the Committee suggest, to be a superintendent nurse of the workhouse or a matron of a separate infirmary, with a medical officer who engages to devote "some of his time to instructing probationers by lectures or otherwise." A qualified nurse, they propose, shall become a trained nurse by serving 18 months at a major training school, and passing the examinations. It is proposed that the term "assistant nurse" shall be dispensed with as misleading. Another important recommendation is that no "trained nurse" shall be appointed superintendent nurse immediately on the completion of her probationership, nor until she has served at least a year as a "trained nurse." Every superintendent nurse, it is suggested, shall be required to hold a midwifery certificate; and further proposals made are—that in smaller workhouses the post of nurse and matron shall be combined; that in country workhouses a slightly higher salary shall be offered "to compensate for the monotony of the life," and that nurses who can acquire but little experience in the infirmary itself, owing to the small number of beds, shall be allowed "to combine a certain amount of nursing of the outdoor poor with their nursing of the indoor poor." With the object of rendering the service more attractive, it is proposed that each nurse shall have a separate bedroom, three weeks' holiday during the year, a day a month, alternate Sundays, half a day a week, and two hours a day off duty. At the first glance it appears to us that if the recommendations of the Committee are carried into effect by the Local Government Board, the supply of nurses might be temporarily greater, but the ultimate result would be the degradation, instead of the elevation, of the standard of nursing, not only in workhouse infirmaries but throughout the country.

### CHRISTMAS PREPARATIONS AT GUY'S.

At Guy's Hospital, where Martha Ward is celebrated for its Christmas party, preparations are busily going forward. "Sister Martha" (Miss Studdert) has already bought a quantity of toys for the tree, and is engaged in thinking out her plans for making the party as great a success as ever. The house surgeon will again personate Father Christmas, and distribute the gifts, and it is expected that either Punch and Judy, or an exhibition of marionettes, will form part of the programme. The date is fixed for the Saturday following Christmas Day, and, as usual, the children of the staff employed about the hospital will be invited, as well as the little patients from the other wards, and the children of the medical staff will also be among the guests. The students will give an entertainment in the wards on Christmas Day, and on Tuesday, December 9th, there will be a vocal and instrumental concert in the Court Room at 8 P.M. This is under the auspices of the Guy's Hospital Nurses' League and Students' Glee Club.

### ROYAL BRITISH NURSES' SETTLEMENT FUND.

A large number of pretty and useful things were for sale on Tuesday and Wednesday this week at the offices of the Royal British Nurses' Association. They were all made by the nurses of the Association, and were sent in for the bazaar held in Park Lane last February, but not sold; indeed, some were not shown, as there was not room for them on the Nurses' Stall. The President, Princess Christian, who had kindly consented to preside, arrived at three o'clock on Tuesday afternoon. A special marquee had been erected in the open space outside, and at the door the President was received by the honorary officers of the Association. She went at once to the office upstairs, where half a dozen stalls had been arranged; and it then became a point of honour among the friends of the Association to try and get near enough to her Royal Highness's stall in the bow window to buy something from it. The stall contained a sample of all the various kinds of work sent in; and on the other stalls were woollen clothing, charity gifts, and art productions—beaten copper, sketches, screens, etc. A number of friends of the Association were present, including Miss Wedgwood, Miss Thorold, Mrs. Dacre Craven, Mr. Langton, (Hon. Treasurer), Dr. Comyns Barclay (Hon. Secretary). Mrs. Langton and Mrs. Dyce Duckworth assisted Princess Christian at her stall.

### NURSES PRESENTED WITH CERTIFICATES IN CHURCH.

When the probationers at the Hospital of the Protestant Episcopal Church in Philadelphia, receive their certificates, or, as they are called in the United States, their diplomas, there is a religious ceremony in the hospital chapel. We have before us a copy of the form of service used on a recent occasion. Fifteen nurses were presented to Bishop Whitaker by the superintendent who said, "Reverend Father in God, I present unto you these women, whom, having finished their course in the Training School of the Hospital of the Protestant Episcopal Church, in Philadelphia, I commend unto you for their diplomas as qualified nurses." The Bishop, addressing the nurses said, "Beloved, we have good confidence that you who are now presented unto us are competent for the work of caring for and ministering unto the sick and suffering, as has been certified unto us by those who have instructed you." A special prayer for the nurses was a feature of the remainder of an impressive function, the presentation of the certificates being followed by a brief address from the Bishop.

### DEATH OF A FRIEND OF THE NURSING MOVEMENT.

Lady Dale, whose death recently occurred at Eastbourne, was a great and generous friend of the nursing movement. She was instrumental in establishing a branch of the Queen's Jubilee Institute at Consett five years ago, and acted as president. There is now a staff of five nurses attached to the association. The deceased lady married Sir David Dale in 1888, was also for some years a member of the Darlington Board of Guardians, and took a deep and active interest in the work. She will be much missed in that part of the county of Durham where she identified herself with every good cause.

### CHANGE OF TITLES AT HAMPSTEAD WORKHOUSE.

With a view to the improvement of the administration and raising the status of the nursing at the Hampstead Workhouse, certain suggestions made by the nursing committee have been adopted by the guardians. Miss K. Platt, who was appointed superintendent nurse last March, is now to be designated superintendent of nursing, the head night nurse is to be called night sister, and the charge nurses sisters. Assistant nurses will be styled nurses. One of the guardians thought it was ridiculous that Miss Platt should be known locally as superintendent of nursing, and reported to the Local Government Board as superintendent nurse. Dr. E. Claude Taylor, medical officer, pointed out that whereas "superintendent nurse" was a term which indicated a kind of forewoman, where all the nurses were more or less on an equality, Miss Platt occupied quite a different position. He held that the change should be made, and added that Dr. Downes, of the Local Government Board, had said that the guardians might use any terms they liked. The suggestion was adopted, but the proposal to call the sick wards the Infirmary was unanimously vetoed. Twenty nurses—the present staff—will be affected by the change of titles. There will be eight sisters, including the head night sister, and the seven "charge nurses;" and as the present "assistant nurses" leave, probationers, whom it is intended to train, will be appointed in their place. Midwifery and the L.O.S. examination will be included in the training.

### QUEEN'S NURSES AT GOSPORT.

The satisfactory report presented at the annual meeting of the Gosport Victoria Nursing Society, established five years ago as the memorial of Queen Victoria's Diamond Jubilee, is from a financial point of view in striking contrast with that of the Brighton Association on which we commented last week. Without any of the wealth of Brighton, the little town of Gosport contributes all that is necessary to enable the nurses to tend 413 cases and pay upwards of 10,000 visits in twelve months; and the balance in hand is £37 8s. 11d. Moreover, not only the public, but the patients also, help to support the movement from which they derive benefit. It is an interesting detail that while two years ago the patients contributed £4 5s. 6d., the amount rose last year to £7 16s. 6d., and this year has reached the substantial sum of £17 2s. There are at present three nurses, about half the number maintained in the huge borough and district of Brighton, and it is in contemplation to appoint another. We have little doubt that Gosport will prove equal to the task of finding the wherewithal.

### MIDWIVES AT THE STEYNING UNION INFIRMARY.

It is creditable to the authorities of Steyning Union Infirmary that at the November L.O.S. Examination, four of the nurses, Misses A. M. Brown, C. Brown, M. Anscombe, and W. Higgins, were successful. The entire staff only consists of the superintendent and ten nurses. At Steyning the policy of the superintendent nurse is to encourage the probationers in their third year of giving them a thorough obstetric training, and sending them up for the L.O.S. examination. She finds that those who are fit to train as midwives, work and study better the first two years in order that they may be allowed to take the maternity wards and lectures.

### OUR CHRISTMAS DISTRIBUTION.

We have to acknowledge, with many thanks, the receipt of parcels of useful clothing as follows:—Nurse Knight, 42 Anson Road, Tufnell Park, N.; A. E. C.; Policy Numbers 370 and 1,240; One of the First Thousand; H. C. Grist, Penrith; E. N. Williams, 15 Alva Street, Edinburgh; Miss Henstowe, Preston Vicarage, Weymouth. All contributions should be directed to the Editor of The Hospital, 28 & 29 Southampton Street, Strand, London, marked "Clothing Distribution." We again remind our readers that the latest day for sending in parcels is Monday, December 15th.

### PROGRESS IN INVERNESS.

The feature at the annual meeting of the Inverness branch of Queen Victoria's Jubilee Institute, was the proposal of Mr. James Anderson that the operations of the nurses should be extended to the country. Mr. Anderson said he was sure that if the County Council were approached they would gladly welcome the extension in county districts, and as he is senior county clerk he ought to be in a position to know. The record of the operations of the branch for the past is admirable from every point of view. The nurses attended 561 cases and paid 6,389 visits. Contributions from patients amounted to £25 as against £19 the previous year, and a sale of work yielded the substantial sum of £34 to the funds.

### A CHARWOMAN AS "NURSE."

In the course of the evidence before the magistrate at Southwark Police Court in support of the charge against George Chapman, who is accused of killing and slaying Maud Eliza Marsh, a woman named Jessie Toon, of 23 Eltham Street, Borough, said she was engaged by Chapman to nurse the deceased. At the inquiry before the coroner it was stated that "the nurse had seen the husband make injections," without a word being said that he acted on the instructions of the doctor. It now transpires that the person in question was not a nurse but a charwoman. Of course no trained nurse would have permitted such an irregular proceeding, but it is as well that the actual occupation of the woman should be known.

### A DUCHESS ON INDISPENSABLE ACCOMPLISHMENTS.

The Duchess of Sutherland has added to the already numerous qualifications which must be possessed by a trained nurse. In the opinion of her Grace, who has been giving an address at Dunrobin Castle on practical points affecting the work of nurses, their training is not complete unless they know how "to make a doll out of a pocket handkerchief, or boats and frogs out of paper." Of course, the Duchess had in mind the nursing of children, who certainly appreciate "the useful accomplishments," which she insists are indispensable.

### SHORT ITEMS.

The *Arundel Castle*, which arrived at Southampton last week, had on board Nursing Sister E. M. Halliwell, of the Army Nursing Service Reserve, and the *Syria*, Nursing Sister E. Hare-Scott, invalided.—An entertainment was given to the in patients of the Cancer Hospital, Fulham Road, S.W., on Thursday last week by the members of the Pinero Dramatic Club under the management of Mr. Adrian Noble.

### Lectures to Nurses on Medical Nursing.

By Eveline A. Cargill, M.D.Brux., L.R.C.P.&S.Ed.

### LECTURE XII.—INFECTIOUS FEVERS.

Fevers are of two kinds, infectious and non-infectious. The infectious fevers are caused by the introduction into the body of a poison, generally a micro-organism, and they run a definite course, the fever being in all cases the most prominent feature.

Fever, whether infectious or not, always consists of the same group of symptoms, showing that every function of the body is more or less disturbed. There is the rise of temperature, hot, dry skin, and flushed face, the tongue furred and moist at first, later dry, brown, and cracked; loss of appetite, thirst, delicate digestion, bowels generally constipated; the pulse rapid, full, and bounding, 100 to 120 or more; later, as strength fails, it becomes quicker and weaker; the respirations hurried, 30 or 40; the urine scanty and high-coloured, sometimes containing albumen; the nervous system disturbed, with headache, dulness, sometimes delirium which may be severe; the muscular system shows general weakness with tremors of lips and tongue and twitching of muscles.

The human body has an average mean *temperature* of 98·4° F., and this is called the *normal* temperature. It varies a little even in health, being higher in the evening and lower in the morning. As human temperature cannot rise above 110° or drop much below 95° without life becoming extinct, the clinical thermometer only registers between these points; and the thermometer is the only reliable test of temperature; neither the patient's sensations or the feel of the skin can be trusted. In order to register correctly the bulb of the thermometer must be completely surrounded by skin or mucous membrane, so we place it in the arm-pit or groin, or inside a cavity, as the mouth or rectum. A cavity temperature is always a little higher than skin temperature, and also more accurate, so where accuracy is of importance it is better to take the temperature in mouth or rectum; in the latter the bulb must be passed in 1½ inch. Cheap clinical thermometers are not reliable, a good accurate one should always be used, and a nurse should be careful always to shake it down before putting it away, and to look at the register before using again. When the temperature is raised to 102° or higher there is said to be "high fever," where it reaches 105° or more there is "hyperpyrexia," while if it fall to 96° or lower there is danger of "collapse."

For the treatment of fever, the patient must rest in bed in a thoroughly ventilated room. Fever patients do not catch cold readily and should be kept as cool as possible without getting chilled, with light bed-clothes. The diet should be light and nutritious, chiefly milk, which must be given in definite quantities at regular intervals. Stimulants are generally required if the fever runs high or lasts long. When the temperature is high it may be reduced by certain drugs, and by the external application of cold; the methods of applying cold being by sponging with tepid or cold water, the wet pack or the cold bath. For the wet pack one or two blankets are spread over bed and pillow, and over them a sheet wrung out of cold water; the patient lies on the sheet which is folded over him and tucked in round neck and feet and then two or more blankets are put on the top and tucked in; the duration of the pack is from ten minutes to an hour. A more vigorous treatment is the cold bath; this should be prepared tepid, about 90°, and after the patient is carefully placed in it, cold water or ice added till the temperature falls to 60° or lower, the duration of the bath being from 10 to 30 minutes; the patient's temperature should be taken while in the bath, and he should be taken out when it reaches 100°, or immediately if a sudden drop occurs or there is any shivering; he is rubbed dry and put back to bed. A stimulant should be given before the bath and hot blankets and bottles ready in case of collapse after it.

The *contagion* of infectious fevers is due to minute living organisms which are given off from the sick to the healthy; these micro-organisms after gaining access to the body require a certain time to establish themselves, and this period during which no symptoms are apparent is called the period of *incubation*, at the end of which (the time varies in each fever) symptoms appear and the *invasion* or onset of the disease itself. The invasion of most of the infectious fevers is much the same; it is sudden, with rigors, vomiting, headache, and high temperature, and after some days the rash or some other characteristic symptom appears.

Though many persons may be exposed to infection only a certain number will take the disease, but as we do not know why some people should be more susceptible to contagion than others, we can only protect all alike by *isolation* of the patient, and *disinfection* of everything likely to carry infection. These methods for preventing the spread of disease are all-important to the health of the community, and the strict carrying out of the details required to make isolation and disinfection efficient must be attended to in nursing a case of infectious fever in addition to the treatment of the fever itself. The nurse has to think not only of her patient, but also how to keep the patient from being a source of danger to his surroundings. For isolation of the patient a separate room is required, if possible, on a separate floor of the house, and shut off from the outside by a sheet wrung out of carbolic lotion (1 in 20) and kept wet. All superfluous carpets, curtains, etc., should be removed, and thorough ventilation kept up. The nurse should wear dresses of washing material, which can be changed before going among other people. Food must be destroyed on leaving the sick-room. Entirely separate crockery, glass, etc., are to be kept for the patient's use, and only such books, toys, etc., be admitted as can be destroyed afterwards. The room is to be kept scrupulously clean, the floor not swept but mopped over with a damp cloth wrung out of solution of chloride of lime (a teacupful to two quarts of water) or perchloride of mercury (1 in 500). All linen used by patient or nurse should be soaked in strong disinfectant before going to the laundry, and woollen things and bedding be subjected to dry heat, 180° to 200°. The excretions of the patient, especially in typhoid and cholera, must be mixed with strong disinfectant (perchloride 1 in 500) and left for some time before being thrown away, and the drains washed down with a similar solution or sulphate of iron (1 ounce to 1 pint of water). When the patient is convalescent and before mixing with others), both he and his nurse must be thoroughly disinfected by taking a carbolic bath (1 in 60), the hair bathed in a stronger solution (1 in 20), and then washed with warm water, the nails require special attention. Entirely fresh clothing should then be put on which has not been in the sick-room.

To disinfect the room sulphur should be burnt in it, a pound being sufficient for an ordinary sized room, placed in an iron vessel over a dish of water. This is ignited with some live coal after the room is rendered air-tight by pasting strips of paper round the window sashes, and wherever air could get in, closing the register of fireplace, or stuffing up the chimney with paper. All cupboards and drawers must be left open, and the contents hung on ropes across the room. Then the door should be locked and key taken out, paper being pasted outside the keyhole and round the door frame, and the room left undisturbed for 12 hours. At the end of this time all windows can be opened and the room thoroughly ventilated; the floor and all paint and woodwork scrubbed with chloride of lime, the paper stripped off and burnt, and the ceiling white-washed.

### Queen Alexandra's Royal Naval Nursing Service.

The Lords Commissioners of the Admiralty inform us that henceforth the following regulations are to be observed for the staff of nursing sisters:—

Qualifications.

1. Candidates for appointment as nursing sisters must produce certificates of training for at least three years at a large civil hospital in the United Kingdom, in which adult male patients are received for medical and surgical treatment, such hospital being also provided with a matron and staff of nursing sisters. Candidates must be of British parentage or naturalised British subjects.

If any doubt arises upon this question the burden of clear proof will rest upon the candidate.

2. The limits of age for appointment will be, not under 25 and not over 30.

3. All appointments, transfers, and dismissals will be dealt with at the Admiralty, as well as all questions in regard to the number of head or nursing sisters to be employed at each hospital. Nursing sisters resigning their appointments are to give three calendar months' notice.

4. Head sisters will, as a rule, be appinted by selection from the list of nursing sisters.

5. All nursing sisters will be required to undergo 12 months' probation before they are confirmed in their appointments. Should they then be reported upon as fit in every respect for H.M. Service they will receive an appointment signed by H.M. Queen Alexandra, and their seniority will count from date of entry.

Pensions and Age Limit.

1. A head sister or a nursing sister shall be eligible to retire on pension after 10 years' service, if she is rendered unfit for hospital duty through disease or injury certified by two medical officers specially appointed to examine her.

2. A head sister shall not continue in the service after the age of 55, nor a nursing sister after 50; and both head sisters and nursing sisters may be called upon at any time to retire on the pension or gratuity earned by their service, should such a course be held to be desirable on public grounds by the Lords Commissioners of the Admiralty, whose decision will be final.

3. A head sister pensioned for disability under the age of 55, or a nursing sister under the age of 50, shall be liable, should such disability cease, to be called upon to re-enter the service with the salary to which she would have been entitled had she not been disabled, and should she refuse so to re-enter the service her pension shall cease.

4. The pension of a head sister or nursing sister shall be calculated solely upon her salary, and the rate shall, after ten years' service, be 30 per cent. of her salary for the preceding year, and shall rise 2 per cent. of her salary for each additional year's service, to a maximum of 70 per cent. of her salary for the year preceding the grant of the pension.

5. A head sister, or a nursing sister, in any case of special devotion to her duties, may be granted a higher pension than that fixed by paragraph 4, but not, however, exceeding £50 a year in all in the case of a nursing sister, or £20 a year in addition to the ordinary pension by service for a head sister.

6. A head sister or a nursing sister disabled in the service, after five, but under 10 years' service, shall be granted such rate of pension below that fixed in paragraph 4 as may be determined by the Board of Admiralty. If she has served for less than five years when disabled, she shall receive a gratuity, to be determined in like manner.

7. A head sister or a nursing sister, retired on account of unfitness for the duties of her appointment, may, provided she has not been guilty of misconduct, be granted a gratuity of one month's pay for each year of service, if not entitled to pension under paragraph 4.

8. In cases where, after a period of not less than six months' sick leave, a head sister or a nursing sister is pensioned for a disability not permanently unfitting her for duty, the pension shall cease on the date when she again becomes fit for duty, unless there should then be no vacancy, in which case, should she be willing to continue her service, she may remain on pension for a period not exceeding one year, pending a vacancy.

9. A head sister or a nursing sister retiring, without having previously obtained permission to do so, or being dismissed for misconduct, shall forfeit all claim to pension or gratuity.

Note.—the regulations as to pension and retirement apply only to nursing sisters entered on or after February 9th, 1901. Nursing sisters entered before that date may be retained until the age of sixty, and their rate of pension will be calculated on the scale allowed by the regulations under which they entered the service.

Salaries and Allowances.

*Head Sisters.*

At Haslar, Plymouth, and Chatham, £125 to £160 by annual increments of £5.

At Malta, none borne, but an allowance of £10 a year will be made to the sister acting as head sister for the time being.

At Dartmouth, none borne.

*Nursing Sisters.*

£37 10s. to £50, by annual increments of £2 10s.

Each head sister and nursing sister at home will be allowed, in addition, 15s. a week in lieu of board, and for the washing of her personal linen, except at Dartmouth, where an allowance of 19s. a week will be allowed. At Malta each will be allowed 21s. a week in lieu of board and washing. Charge pay to senior sister in charge of hospital ship, 1s. 6d. per diem.

Quarters and Servants.

1. Furnished apartments will be provided for the staff.

These will include a mess-room, reading-room, kitchen, and offices; a sitting-room and bedroom for the head sister; and a separate bedroom for each nursing sister.

Fuel and lights will be provided throughout the quarters.

The mess, which it will be obligatory on all the members of the staff to join, will be furnished with the necessary requisites of plate, linen, glass, etc., and the mess accounts will be audited quarterly.

2. The head sister will be president of the mess, and she will be charged with the duty of maintaining due order and regularity, as also with the general duty of causing the rules and regulations of the hospital to be strictly adhered to in the quarters.

3. Wines or other stimulants are not to be introduced into the sleeping rooms except under medical order in cases of sickness.

4. The nursing sisters are to keep their sleeping rooms in a tidy and orderly condition, and stores belonging to the wards are not to be kept therein.

5. They are not to be absent from prayers or from meals except with the sanction of the head sister.

Maid servants will be appointed for attendance on the

### QUEEN ALEXANDRA'S ROYAL NAVAL NURSING SERVICE.—*Continued.*

staff at each hospital, and they will be borne on the hired list.

Their wages at home will be on the scale authorised by their Lordships, and they will be allowed 1s. 6d. a day in lieu of provisions (which will be paid to the head sister), and 1s. a week for the washing of their personal linen. At Malta the wages will be 2s. 3d. a day without any allowance, board, or uniform.

They will also be allowed annually—

At home: Two print dresses, six white aprons, six white caps, six white collars, which will be washed at the hospital laundry. No uniform allowed abroad.

### Uniform.

1. Each head sister and nursing sister will be provided with the following articles of uniform:

Navy blue surge dresses with scarlet cashmere cuffs, and, above the right elbow, an embroidered badge consisting of a red Geneva cross on a white ground in a gold border, and above, her Majesty Queen Alexandra's monogram, viz., two A's red, interlacing an anchor and cable gold, the whole surmounted by the Imperial Crown, three annually.

Cape to match dress, one annually.

Muslin caps, six annually; plain linen collars, twelve annually; cuffs to match, twelve annually; white aprons, twelve annually. These will be washed at the hospital laundry.

Navy blue straw bonnets, with navy blue ribbons, two annually.

Navy blue tweed cloaks (with sleeves), one summer one winter, biennially.

2. Uniform is always to be worn when on duty within the precincts of the hospital.

3. At Malta, in hot weather, white cotton blouses with the regulation cuffs and badges are to be worn instead of the blue body of the dress, and all nursing sisters are to dress alike. Four blouses allowed annually.

4. Head sisters and nursing sisters dismissed for misconduct, or resigning their appointments, must return all articles of uniform that have not been worn twelve months.

### Official Position and Foreign Service.

The head sister and nursing sisters are to be regarded as officers of the hospital, taking a position immediately after the surgeons, and are at all times to receive the respect due to their position.

The duties of the head sisters and nursing sisters will be limited to the hospitals to which they are attached, but they will be liable to be transferred to other Naval hospitals at home and abroad, as well as in exceptional cases to hospital ships. First-class railway passes will be supplied to them when sent on duty by rail.

### Head Sisters' Duties.

1. The head sister will, in addition to the nursing of her own cases and charge of her own wards, exercise general control and supervision over the nursing sisters, visiting the wards where they are employed at any time she may think proper.

2. She is to allot specific duties to the nursing sisters, distributing them through the wards as may be considered necessary by the principal medical officer; and she is to be careful to see that all the orders and instructions of the medical officers for the treatment of cases are punctually and fully carried out by the nursing sisters.

3. She is also, under the direction of the medical officers in charge of wards, to arrange the detail of night duties for the nursing sisters, and for the sick-berth staff under instruction.

### The Duties of Nursing Sisters.

1. The nursing sisters will receive their instructions from the medical officer in charge of the wards to which they are attached. The medical officer will also determine, as he may judge fit, according to circumstances, the hours at which the nursing sisters are to attend and are to be relieved.

When possible, they will be allowed two hours daily for recreation.

2. They will be assisted in the wards by stewards and attendants of the sick-berth staff, to the latter of whom they will give such instruction in nursing as may be necessary.

3. They will be responsible for the personal cleanliness of the patients who are confined to bed; and for seeing that all medicines, appliances, diets, and "extras" are administered according to the instructions of the prescribing medical officers, whom they are to accompany in their visits to the patients under their care, duly noting the orders that may be given.

4. They are to attend and render assistance at surgical operations when required.

5. They are not to remain in or visit the wards when not on duty unless officially sent.

6. On the death of a patient, the nursing sister of the ward is to communicate the fact immediately to the medical officer of the ward, the head sister, and the ward master.

The ward master will then, in the presence of the nursing sister, the attendant of the ward, and two of the patients, take an account of any articles which the deceased has left, and, after the same has been duly signed by those persons as witnesses thereto, he is to take it with the articles to the store officer.

7. The nursing sisters are to assist in training the sick-berth attendants in practical nursing duties, in the method of handling patients, applying dressings, administering medicines, diets, and "extras," and in sick-room cookery.

8. They are to represent without delay any neglect of duty, or impropriety of conduct, on the part of any of the sick-berth staff or patients, to the ward master, who is responsible for the maintenance of discipline in the wards, as well as for the cleanliness of the wards, passages, staircases, furniture, etc. A report by the nursing sister to the head sister, detailing the facts, is also to be made in all such cases, and this report, together with the remarks of the head sister, thereon, is to be forwarded by the latter to the medical officer in charge of the ward for such action as he may think desirable.

9. If nursing sisters are in any doubt or difficulty, reference is to be made at once to the head sister, who will, if necessary, bring the matter to the notice of the medical officer in charge of the ward, or medical officer on duty.

10. All reports or suggestions which nursing sisters may have to make relating to their duties are to be submitted through the head sister to the principal medical officer, for the consideration of the medical director-general.

11. No member of the staff is at any time to accept a present from a patient or his friends; and any letters received by the nursing sisters from the patients are to be shown to the head sister.

12. The medical officer in charge of wards will, without delay, notify to the head sister any neglect or misconduct on the part of a nursing sister, and, if necessary, he will also report the same to the principal medical officer, who is to forward a full report of the case to the medical director-general.

13. Letters that may be sent to the friends of patients from the head sisters or nursing sisters are not to convey any

### QUEEN ALEXANDRA'S ROYAL NAVAL NURSING SERVICE—*Continued.*

medical opinion whatever on the case, unless such opinion is specially obtained from the medical officer, and is communicated with his sanction.

### Leave of Absence.

1. Provided their services can be spared, a head sister may be granted leave for 42 days in the year, and a nursing sister for 35 days, in both cases inclusive of Sundays. They may be allowed, in addition, an afternoon once in 14 days, and every fourth Sunday from 8 A.M. to 10 P.M.

2. Of this leave, four days at a time may be granted by the principal medical officer; but for any period exceeding four days the approval of the medical director-general will be necessary on the recommendation of the principal medical officer.

3. In the case of the head sister, the application is to state the name of the nursing sister who will undertake her duties.

4. If serving at a hospital outside the United Kingdom, each nursing sister will be allowed 35 days' leave a year, as at home, but with the option of reserving it from year to year for lengthened leave on return to England, provided that arrangements be made without putting the public to any expenses in providing substitutes; the maximum leave reserved being three months.

### Sickness and Medical Control.

In the event of the illness of a head sister or nursing sister, she will be entitled to medical attendance in the establishment, and will be dieted in accordance with the prescription of the medical officer attending her. She will receive her full salary and allowances, but 1s. 6d. a day will be deducted in lieu of provisions, so long as she remains in the establishment as a patient.

If the illness is clearly attributable to the service, no further deduction will be made; but if not attributable to the service, the case will be considered after 42 days, and dealt with according to circumstances.

Nothing contained in the foregoing regulations is to be taken as relieving the head sister and nursing sisters from being subject to the general control and supervision of the principal medical officer of the hospital to which they are attached.

### A Day's Work in a Boer Refugee Camp.

BY A NURSING SISTER.

The refugee camp was situated on the open veld about 15 minutes' walk from one of the large towns in South Africa. The site was not all we could wish, as a military camp had been pitched on the same site previous to our's, but nevertheless we had to make the best of it, and when at last we were able to get E. P. tents, to replace many of the old bell tents, the camp presented a very different appearance.

The people in the camp numbered a little over 4,000, most of these being women. There were, however, about 1,200 children, 800 of whom attended school. Most of the husbands and brothers were prisoners of war in St. Helena, though there were still a good many out on commando, boys of 10 and 12 being amongst the latter. These boys were very good shots; for example, after peace had been declared, among the burghers who returned to the camp was a boy of 12, who had been out on commando during the greater part of the war. He entered the camp in full uniform, riding his pony, with a large haunch of mutton hanging from one side of his saddle and a kettle from the other. He was carrying his rifle, and when asked how he had got on, replied, "Oh! I have killed five or six Englishmen, but I mean to kill some more." Most of the men in the camp were willing to work, some voluntarily and others for money. The policemen, carpenters, shoemakers, dispenser, storekeepers, clerks, and brickmakers (the bricks are made of mud and water, and baked in the hot sun), were all paid so much per diem, while the head corporal, sub-corporals, and rations dealers were voluntary workers.

### Accommodation and Camp Life.

The camp consisted chiefly of bell tents. Some of the more wealthy Boers had their own private square tents, but eventually we had about 72 E. P. tents sent up. Usually 12 people (including children) were allowed to live in an E. P. tent, while not more than six or seven in a bell tent. The camp was divided up into sections, the sections into lines alphabetically of about seventeen tents each, and each tent was numbered.

I had one sister and 16 camp probationers working with me in the camp. The probationers were all Dutch girls, and I made them responsible for about fifty-six tents each. Their uniform was pale pink and white striped galatea dresses, white "cappies" (Boer bonnets, and very picturesque), white aprons, and white bands with a red cross on their left arms. They were each provided with a basket book, pencil, and thermometer. At 7.30 every morning they made their daily round, visiting tent by tent in their several sections. On finding any cases of sickness they immediately took the temperature of the patient and entered it in their book together with the name, age, section, line, tent, and remarks about the case.

### A Day's Work.

At 8 A.M. every morning porridge was served out in the large soup kitchen to all children under 15, old people, and invalids. As a rule there were about 800 names given in. At 9 A.M. the nine probationers belonging to my divisions of the camp came to my office tent to give me their report of the daily visit, after which the doctor came and treated all out-patients. I had in my tent a cupboard in which I kept all medical comforts, so that we were able to treat most of the cases there with the exception of the dental cases, and these we always took across to the dispensary. Many of the out-patients only came for "castor oilee." This is a very favourite medicine, and I have often had to give out as many as 80 doses in one morning! (by a dose I mean ℥j.). One patient would ask for nine doses for her family, another for seven, and so on. I always made it a rule to keep, if possible, five 2-pint bottles amongst my medical comforts. I do not think they always drank the amount given them, for they were allowed to take it home with them, and I know of one or two cases where they used it to soften the leather of their shoes! We treated all first-aid cases in my office, but if there were any serious ones anongst them we sent them to the hospital at 11 A.M. in charge of the camp probationer in whose section they lived. At 10 A.M. anyone might come and see me who required new tents, clothing, blankets, cooking utensils, boots, babies' food, etc., when I took down all names and particulars. After this, if there were any serious cases in the camp, I went with the probationer to see them in order that when the doctor came round with me at 11 A.M. I might be able to give him full details of the case. At 12 o'clock four of the probationers went to the large soup kitchen and gave out soup to about 700 children (under 15), invalids, and old people. I was, as a

### A DAY'S WORK IN A BOER REFUGEE CAMP—*Continued.*

rule, present and helped to check the names on the cards which were given out, newly filled in, the first of every month. We had pink cards for porridge, green for soup, and yellow for fresh milk. A line was drawn through the date daily as they gave up the card for soup, so that the same person could not come a second time in one day. At 2 P.M., when on duty, I always liked to go round the camp visiting tent to tent with the various probationers. It was in this way that I found out for myself any cases of overcrowding, dirty and untidy tents, and destitution. The probationers usually reported these cases in the morning, but I always made it a rule to visit such cases personally before entering them in my books. At 6 P.M., if there were any mild cases of sickness left in the camp the probationer, after taking the temperature, came and reported them to me, and, if necessary, I visited them. At 8 P.M. all outside work was finished for the sisters, also for the probationers (though they were first called if a new case occurred in the night, after which they fetched the doctor), though there was plenty of writing and book-keeping to be done.

### A Letter and an Incident.

Whatever was given out to the people in the way of new tents, stretchers, clothing, blankets, etc., had in the first place to be entered on a small slip with my signature. I then sent it, through the medical officer, to the superintendent, who signed and stamped it. It was then returned to me, and I gave it to the probationer, who handed it to the recipient. All these things had to be entered in the various books, so that no one could obtain the same articles a second time. I often had quite a post of letters from the camp people, asking me for clothing, etc., some of which were most amusing. This is a copy of one, but it is not as funny as some of them.

Dear Sister,—I kindly wanted to request you if you can't give me some clothing please, and I am here nine months already, and I did not get anything yet. I will be very glad if you will supply me with

12 yards zis (print).
9 yards dress stuff.
12 yards flannelette.
12 yards linnen.
4 paar housen (stockings).
2 small paars for a baby.
2 pairs shoes, one of size 6 and one baby's shoes of size 4.

Will oblige your friend,
Mrs. N. H.

Camp, E. Line. Tent No. 9.

This is by no means one of the largest orders I had. At first the people never gave me a minute's peace, for they appeared at my door early morning and continued to come all hours of the day, even during meal hours. Some of them were very grateful for all the British did for them; but one woman, whose husband joined the Refugee Camp Police, refused to cook for him and finally to own him as her husband. I sent for her, and when asked why she behaved like this, replied: "I am not going to have anything to do with a husband who goes and works for the British. Will you please kindly give me some clothes for my children and two blankets?" "Oh! no," I said, if you won't have anything to do with the British you must not expect them to give you anything!" She kept to her word and I to mine.

### Treatment in Sickness.

The Boers are very funny with regard to treatment in sickness. Here are a few instances:—

1. A child is very ill with pneumonia, so a goat is killed and the skin while still warm is wrapped round the body of the child.

2. A favourite poultice is made with the hairs cut from a cat's body and baked in the oven.

3. For chapped or rough hands make an ointment of tallow grease and paraffin. Though the smell is very unpleasant, the ointment is really very good.

4. Soap and sugar plasters are used in place of our boracic fomentations.

5. Tar spread on linen for swollen glands and rheumatism.

6. A steel chain or bracelet worn on the wrist to charm away pain in the arm or shoulder.

It is to be hoped that the probationers, who were very keen on learning all they could, will be able to convince the people in their own homes that the "British" treatment is after all the best treatment. I had very little time for classes but was able to give them a few lessons on the "use of the clinical chart and thermometer, bandaging, weights, measures, signs, and various cases of first-aid treatment.

### A Nurse's Life in a School.

BY A CORRESPONDENT.

Having read several "Nurses' Experiences in Private Nursing," I venture to try and write a little of my own experiences as nurse in a school. Of course I know the general opinion is that in a school there is very little real nursing to be done, and that it does not require actual good training to undertake a post of this kind. That is to a certain extent true: a school is not a place where people are sent who are ill, but only a place where they fall ill accidentally in much the same way as in their own homes. At the same time a person holding this position who has not had some good training would often find herself in a difficulty over some of the cases that come under her notice. One's thoughts at once fly to infectious cases. An epidemic of measles, mumps, or scarlet fever is a very common occurrence in large schools, owing to the fact that young people are so susceptible and come from all parts.

### A Girls' College.

I have recently taken a post as resident nurse in a large girls' college, holding about 190, this including 16 resident mistresses and a large staff of servants. I could not describe the routine of work, as it is very erratic: you cannot tell from day to day how many patients you will have. Small things are always happening; indeed, at times it reminds me of "casualty." The girls come up voluntarily, or are sent with their different complaints, the majority being slight, which a dose of medicine or a bandage will soon put right, and I say "come up again to-morrow." Other cases are not so easily dealt with. I find a temperature or other symptoms that involve detention and nursing. Many bad illnesses are prevented by prompt attention at the very beginning, and certainly the old proverb, "Prevention is better than cure" is well carried out here. Still, as we know, some things will take their course, do what you will.

### Serious Cases.

Last term one of the mistresses had what she thought was indigestion. However, she kept her room. I poulticed her and ordered low diet. Next morning temperature was 101, and the doctor pronounced her case appendicitis. A case of pericarditis happened in much the same way: the child had been kept in bed two days with apparently very little the matter; she was grumbling at being kept from school when feeling so well, an exception to the rule; but a bad heart attack was not to be averted. She was kept in bed several more days, but without the grumbling. I mention these cases to show that good nursing is required, and that all the cases are not small and trivial.

### SHAMMERS AND THE THERMOMETER.

A word about the "old soldiers," that is our term for shammers. Of course one gets to know the regular shammers, and can treat them accordingly; there are others who at the very thought of a disagreeable lesson find that they have a very bad headache. Once a child was heard to say, "I coughed as hard as I could to get some cough lozenges from nurse." Since then the lozenges have been changed to some not quite so palatable, and the demand for them is decidedly less. Often the children describe their feelings in a very funny way, especially if there is nothing much the matter. The thermometer is a great friend of mine, for it can tell me the real from the sham.

### A HAPPY LIFE.

A nurse's life in a school can be a very happy one. It is true that one is rather much alone, but in my case the sanatorium is attached to the school, so that I am brought constantly in contact with mistresses and pupils, I am also invited to the school functions, concerts, teas and dances. There is great pleasure derived from watching and studying young people. I frequently wonder what sort of women some of these girls will make. Undoubtedly nursing in all its branches gives unbounded scope for the study of human nature.

### Appointments.

[No charge is made for announcements under this head, and we are always glad to receive, and publish, appointments. But it is essential that in all cases the school of training should be given.]

BOROUGH ISOLATION HOSPITAL, PLYMOUTH.—Mrs. Helena K. Rapton has been appointed night sister. She was trained at Leeds General Infirmary, and has since been attached to the West of England Co-operative Association, Plymouth; night sister at St. Bartholomew's Hospital, Rochester; and charge nurse at the South-Western Fever Hospital, Fulham.

BRICKLOW UNION, KNUTSFORD.—Miss Ellen Pitt has been appointed assistant nurse. She was trained by the Meath Workhouse Nursing Association at St. Peter's, Maybury Hill, Woking.

CITY HOSPITAL FOR INFECTIOUS DISEASES, NEWCASTLE-ON-TYNE.—Miss Effie Bamford has been appointed superintendent of night nurses. She was trained at the Mill Road Infirmary, Liverpool, and has since been staff nurse at the Hospital for Consumption and Diseases of the Chest, Brompton, and sister at the Borough Sanatorium, Peasley Cross, St. Helens.

CITY SMALL-POX HOSPITAL, BIERLEY HALL.—Miss Elizabeth Martin has been appointed nurse-matron. She was trained at Kensington Infirmary, and has since been ward sister, night superintendent, and temporary superintendent at Bradford Union Hospital, and superintendent nurse at Barnsley Union Infirmary.

FIR VALE UNION INFIRMARY, SHEFFIELD.—Miss Edith S. Butler has been appointed charge nurse. She was trained at the Whiston Infirmary, Prescot. She has also had one year's training at the St. Helen's Sanatorium, and for the last eight months has done private nursing for the Oldham Nursing Association.

HULL CORPORATION SANATORIUM.—Miss E. S. Miles has been appointed sister in charge. She was trained at the Taunton and Somerset General Hospital, where she afterwards took sister's holiday duty. She has since been charge nurse at the North-Eastern Hospital, Tottenham, for five years, and for three months did night superintendent's duty.

JESSOP HOSPITAL, SHEFFIELD.—Miss Annie E. Firth has been appointed sister. She was trained at Stanley Hospital, Liverpool, and at Manchester Maternity Hospital for the L.O.S.

KINGSTON HILL UNION INFIRMARY.—Miss Mary Pritchard has been appointed night superintendent. She was trained at Poplar and Stepney Sick Asylum, where she remained for two years as sister. She has been relief sister at Kingston Union Infirmary since October last.

LAMBETH INFIRMARY.—Miss Lily May Candy has been appointed sister. She was trained at the Gloucester Royal Infirmary, where she was afterwards charge nurse for two years, and private nurse for one year. She has lately been staff nurse at Sheffield Royal Infirmary.

OPHTHALMIC SCHOOL, HANWELL, W.—Miss Nellie Thornton has been appointed charge nurse. She was trained at St. Bartholomew's Hospital, London, and has been for the last two years at the Hanover Institute, W.

ROYAL CORNWALL INFIRMARY, TRURO.—Miss Mary L. Parker has been appointed ward sister. She was trained at the General and Eye Hospital, Swansea, and has since been junior sister at the Royal Victoria Home, Bristol, and sister *pro tem.* of the Male Medical Wards at Swansea Hospital.

SKINNINGROVE MINERS' HOSPITAL.—Miss E. Elgey has been appointed matron nurse. She was trained at Stockton and Thornaby Hospital, and has since been district nurse at Middlesborough and elsewhere. She has also been engaged in private nursing.

SOUTH-EASTERN FEVER HOSPITAL, NEW CROSS.—Miss Louise F. Woodnutt has been appointed charge nurse. She was trained at the Central London Sick Asylum (Hendon Branch), and was charge nurse at the Long Reach Isolation Hospital, Dartford, Kent, during part of the small-pox epidemic.

WEST DERBY UNION, LIVERPOOL.—Miss Janie Teresa Gore has been appointed charge nurse. She was trained at Brownlow Hill Infirmary, Liverpool, and has been attached for two years and a half to the Nurses' Institution, Aylestone Road, Leicester.

### "The Hospital" Convalescent Fund.

THE honorary secretary acknowledges with thanks the receipt of 10s. from "A Hospital Sister," 10s. from Miss E. M. Williams, and the receipt of 1s. from Nurse M. Ruel, and thanks her for her kind promise to subscribe 2s. 6d. annually.

### To Nurses.

**WE invite contributions from any of our readers, and shall be glad to pay for "Notes on News from the Nursing World," or for articles describing nursing experiences, or dealing with any nursing question from an original point of view. The minimum payment for contributions is 5s., but we welcome interesting contributions of a column, or a page, in length. It may be added that notices of appointments, entertainments, presentations, and deaths are not paid for, but that we are always glad to receive them. All rejected manuscripts are returned in due course, and all payments for manuscripts used are made as early as possible after the beginning of each quarter.**

### Everybody's Opinion.

[Correspondence on all subjects is invited, but we cannot in any way be responsible for the opinions expressed by our correspondents. No communication can be entertained if the name and address of the correspondent are not given as a guarantee of good faith, but not necessarily for publication. All correspondents should write on one side of the paper only.]

### A CORRECTION.

"Miss Mary F. White" writes from St. George's Infirmary, Fulham Road, S.W.: Will you kindly correct the error with regard to my training which appeared in the "Appointments" of last week's Hospital? My three years' certificate was gained at St. George's Infirmary, Fulham Road, not at Leicester Infirmary. Therefore, the appointment should read "probationer and head nurse at St. George's Infirmary, Fulham Road." I also hold the L.O.S. certificate.

[The particulars were printed as supplied by the authorities at Aylsham Workhouse.—Ed. Hospital.]

### A DISCLAIMER.

Miss Frances H. Low writes: As your journal is so widely read by nurses, will you have the goodness to allow me to make this disclaimer in the hope that it will reach the majority of them? I have just learned, to my great amazement and vexation, that an article on "Hospital Nurses" which appeared a few months ago in the "Nineteenth Century," under the name of M. Johnstone, is attributed by a large number of nurses to myself. If, therefore, you will allow me to assert in the most deliberate and emphatic manner that I am not the author of that article, and do not hold the opinions and sentiments there expressed, I shall be extremely obliged.

### THE CORONATION NATIONAL FUND FOR NURSES IN IRELAND.

"Another Dublin Nurse" writes: As one of the Dublin Nurses I desire to make a few remarks in reply to the letter of "A Dublin Nurse" and the editorial comments upon said letter which appeared in The Hospital for November 22, in reference to the Coronation National Fund for Nurses in Ireland. There is no intention to start a Separatist Fund. Rather it is intended by the movement to make some provision for our 3,800 nurses in Ireland who, owing to the small salaries which they receive have not the means to spare the amount which would enable them to pay the premiums of that excellent institution, the Royal National Pension Fund of London. For while there are about 4,000 female nurses of the sick in Ireland certificated and uncertificated, yet there are not more than 140 of these nurses who have been able up to the present date to take out pension policies in the Royal National Pension Fund since its foundation in 1887; and there are over 6,000 in England and 300 in Scotland who hold policies. By the movement made in Ireland there is no intention to rival even in a small way the Royal National Pension Fund. Judging by what has been said by some of the persons who have advocated the founding of the Nurses' Fund here, it is intended rather to supplement the good work of the great English fund. But while such excellent provision is made for the 140 who have large salaries, why should not the effort be made by the friends of the nurses in Ireland to make some provision for the 3,860 in time of distress? The movement is now supported by most of the Dublin nurses, who have elected from amongst their body four matrons to confer with a small sub-committee appointed by the Citizens Committee. Amongst the latter are Sir William Thompson, C.B., Surgeon in Ordinary to his Majesty the King in Ireland, and Sir Christopher Nixon, ex-President of the Royal College of Physicians in Ireland. The principles upon which the fund should be worked are being considered by this joint committee, so that the representatives of the nurses will have a voice now in forming the rules, and hereafter share in the management of the fund. It is intended first to start with a benevolent fund for aiding nurses in need of help in time of lengthened illness, and, if the fund grows sufficiently large, to grant pensions to those most in want of it. It is also intended to have a provision made in the rules by which aid may be given to the members of the Irish society to take out policies in the English fund. The subscription qualifying a nurse to join the society will be small. I am sure, Mr. Editor, as we know you to be a kind-hearted man, you would rather encourage than discourage this effort, though it be small in dimensions as compared with those of the wealthy Royal National Pension Fund; but kindly remember that the people in Ireland are comparatively poor as well as the nurses who work here and with whom the public sympathise. I am an Englishwoman, but have been working in Ireland for some years. The feeling is growing amongst nurses in Dublin that the time has come when they should form some definite organisation amongst themselves, and I feel sure that this benevolent fund will provide a means by which this object may be obtained. I must say I sympathise, and believe this to be an excellent scheme for nurses in Ireland.

### NURSING HOMES IN MARYLEBONE.

"D. H." writes: I know nothing of the alarming document referred to in your issue of November 8 beyond the extracts you gave, but judging from these I must entirely demur to the justice and reasonableness of it. It is true that many of these homes are established and maintained and apparently flourish in the parish. The necessities of the case almost drive them to this limited area, for to fulfil their purpose they must be within easy reach of the medical specialists who congregate chiefly in Harley Street and its vicinity, and who are far too busy to visit homes scattered further afield. Now a nursing home is an exceedingly expensive institution to establish and maintain. Leases in Marylebone are difficult to acquire; rents, rates and taxes are high; salaries and wages are ever on the increase. The very fact of the multiplication of these expensive homes proves that there is a demand for them, and surely as the demand exists it is only natural and right that it should be met by a commensurate supply. As to the specific charges made I presume the petitioners are prepared, or think they are, to prove them. Personally, and I think I know what I am writing about, I cannot conceive of "noxious odours and distressing sounds" penetrating beyond the house walls. Within doors even patients and their friends and their medical men would instantly and loudly complain if either were so perceptible as to cause annoyance. I agree that a nuisance is occasionally caused by the laying down of straw in the streets, but surely for this the sanitary authority is responsible, and this protest is not, I presume, addressed to the Borough Council. Again, I cannot see that the residents of Marylebone, as such, have any concern with the social status or technical training of the staff of these homes. Any protest against the internal management of an entirely private business seems to me to be an impertinent interference. In any case, the allegation that housemaids and ex-barmaids are commonly employed as nurses in nursing homes is, to anyone knowing the real facts, too absurd. A home so conducted in Marylebone would speedily be heard of in the Bankruptcy Court. A surgeon or physician is only too ready to criticise even highly skilled nursing, and I tremble to think what any specialist would say if asked to entrust one of his patients to the care of a "housemaid or ex-barmaid." I know of cases where doctors, for the sake of economy, have sent their patients for treatment or operation into Marylebone lodging houses—houses which make no pretence to hygienic management or any particular comfort or convenience for invalids, and where, no doubt, the attendance is largely undertaken by the maid-of-all-work. But these are not nursing homes, and the temporary use of them as such is strongly to be deprecated. I have read something, too, in the newspapers of the management of certain so-called nursing homes in the suburbs, where patients are nursed somehow for sums under £1 a week. If the registration you mention would defend the nursing homes of Marylebone against charges possibly deserved elsewhere, I am certain that their owners would most warmly welcome it. This protest comes, I gather, from some private residents of Marylebone. I wonder if the tradespeople, ratepayers like themselves, would endorse their appeal?

"Resident" writes: Perhaps you will be so good as to allow me, as one of those who approved of the protest against the multiplication of nursing homes in this parish, the privilege of a rejoinder to the letter of "Yorkshirewoman." Her reply we think more impulsive than logical; it is discursive, and does not disprove any of our allegations. We are taken to task for exercising our right as residents of from 20 to 30 years in this parish for complaining of evils that we know exist. How irrelevant it is to remind your readers of the noble sacrifices made by nurses; all honour to the latter! We spoke of certain "nursing homes" in this parish, institutions personally known to us, wherein were domestic servants, former barmaids, etc, and without hospital training, wearing nurses' uniforms. It is common knowledge that this abuse exists, and it is idle to deny it. Moreover, it is no secret that such institutions have time and again admitted infectious cases, and two such "homes" are known to us in which those cases have within the last year been admitted. As has been well said, "there is a backstairs freemasonry, and through the maids things leak out." "Yorkshirewoman" does not apparently understand why the "noxious odours" of which we complain are so often objectionably noticeable: they come mainly from houses intended as private residences, and these are in consequence structurally unfit for conversion into hospitals, and have not the conveniences for the proper and sanitary disposal of hospital refuse. She unconsciously uses one of the most cogent arguments against the introduction of nursing homes in residential quarters when she inveighs against the alleged selfishness of private residents, for obviously it would be better to establish nursing institutions in quarters where the minimum amount of annoyance can be caused by and to private inhabitants. It is well known that there are in this parish several streets containing so many nursing homes that the proportion of the latter to private residences is as four is to one. Hence our well-founded complaint against the multiplication of nursing homes. A word anent "Yorkshirewoman's" remarks as to the frequent laying down of straw: the Medical Officer of Health for Marylebone in a recent report says: "The frequent complaints about this straw laying show that it is becoming a nuisance of some magnitude; that what may be called the nursing streets are almost permanently covered with straw; and that, apparently, there do not exist sufficiently definite powers to deal with the litter or straw until it becomes offensive." I have fortunately preserved the article that appeared in your excellent journal about two years ago. You say, commenting upon that report: "It is not entirely without reason that the residents in certain districts of the West End object to houses in their streets being turned into nursing homes. Not that we need have any sympathy with the more or less sentimental objections which are sometimes raised to the continued propinquity of sick people. Still, when houses are close together the sick are of necessity a nuisance to the healthy. That one's piano should be hushed, that sounds of revelry by night should not be permitted to arise, and that one should live in a state of perpetual consideration for the feelings of one's neighbours are things which, though distinctly disagreeable, one puts up with, with such grace as may be possible when a bonâ fide neighbour is seriously ill. We do for others what we expect to be done for us. The matter, however, is rather different when a constant series of sick people are imported into a street for purely business purposes." I trust that you do not think my letter too lengthy; the subject, however, is so important to the inhabitants of a large area in the West End that you will perhaps kindly excuse the length of this communication.

### CHELTENHAM GENERAL HOSPITAL.

"Mr. H. T. Carrington, honorary secretary and treasurer of Cheltenham General Hospital," writes: I hope you will allow me to correct a misleading statement relative to the question of the certificates and testimonials of the dismissed nurses which appeared in the correspondence of your paper of November 29th. No message was sent by the committee to the 14 nurses that unless they apologised they would forfeit their certificates; indeed, it was from the first the intention that they should receive these, as a matter of course. With regard to testimonials, I am not aware that a general testimonial has ever been given to any nurse on the private staff (certainly, none has been issued from the secretary's office during the six and a half years I have held the post), as with the nurses in question a reference could always be made to the matron by any nurse seeking another post. In short, as regards certificates and testimonials, all the private nurses who have left, whether of their own free will or upon notice, have been treated in the same manner and stand on the same footing.

### ASYLUM ATTENDANTS.

"Injustice" writes: I have read with great interest the different opinions expressed regarding the training of the asylum nurse. I would like to ask what inducement is given to nurses to train if, when having gained their certificate, and a better position is to be obtained, they find it given to a nurse who holds no certificate at all? I know that in a large county asylum in Kent two nurses hold the position of head attendant, neither of whom has a certificate nor has had very many years' experience of mental nursing. This promotion took place recently, and at the time there were several nurses in the institution who possessed the Medico-Psychological certificate, who had been there a great many years, and who, naturally enough, expected the vacancies to be offered to them. I may add that similar promotion took place on the male side. Is such a thing right or just? Nurses are told that they must train, but is it likely that women of refinement and education will take up mental nursing when such injustices are done? Moreover, it must be very hurtful to a trained mental nurse to have orders given her by a person placed in authority, who really knows less about how things ought to be done than she does herself. How, too, can nurses respect such people when they are placed in authority over them?

"A Reader" writes: I have read with much interest the letters, *re* asylum nurses, which have recently appeared in The Hospital. I fully agree with those writers who think that the asylum nurse's work entitles her to more respect than she at present receives. Do you not think that if the nurses were drawn from a superior class, and conducted themselves in a manner calculated to inspire respect that they would receive it? I do not wish to say one word against asylum nurses, but at the same time one cannot shut one's eyes to facts, and the very patent fact that stares us in the face is that the majority of women employed in asylums are selected for their physical qualities. I think that superintendents and those responsible for engaging nurses should look further than this, and let tact, education, and aptitude for mental nursing weigh with them when choosing a nurse. I myself know several asylum nurses whose lack of education is painfully evident—the writing of their names being regarded as a task of no little magnitude. Yet these are employed and put on the same footing as really superior women, who, by the mere fact of their presence in an asylum, and conscientious discharge of duty, unconsciously raise the standard of mental nursing. In a large public asylum in the north, the nursing staff is largely drawn from kitchen and scullery maids; and as soon as it becomes known that a nurse is about to resign, there is a flutter amongst the domestic staff, some members of which at once apply to be "nurse." If she is sufficiently "sizeable," the superintendent and matron approve, and she is straightway transplanted from the congenial region of pots and pans, and the kitchen knows her no more. Surely this is not the way that the social position of the asylum nurse is to improve. One would think, too, that "nurses" taken from such a source would occupy humble positions in the wards; but no, once in the wards the ascent is rapid and sure, and in a very short time they fill positions of importance, such as charge and deputy charge nurses, often being promoted over the heads of nurses who have been years longer in the house, and who originally came as nurses.

### OUTDOOR UNIFORM.

"An Army Nurse" writes: The question of outdoor uniform is one of perennial interest. Uniform has very much to recommend it, being economical, easily put on, and last, but by no means least, decidedly becoming in most cases. One would wonder in the face of this favourable evidence what could possibly be said against the wearing of outdoor uniform, but it must be remembered that "it is not the cowl that makes the monk" and in these days cowls are too often donned by persons whose only monkish habits are those they wear upon their backs. In other words, many people adopt nurses' uniform with no title whatever to do so. Naturally this disgusts the rightful wearers of the neat bonnet and cloak which at one time always commanded respect as being the badge of an honourable and useful calling. Some nurses belonging to a large and well-known institution have been considerably annoyed lately by a couple of nursemaids dressed in a kind of uniform just the same colour as their own, who came morning after morning, and hung about the gates carrying on a brisk flirtation with the men on duty there. Any casual observer might easily jump to the conclusion, seeing these young women dressed like the hospital staff, that some of the nurses were amusing themselves in this questionable manner. One really cannot blame the maids, they do not choose their garments, but mistresses who cause their servants to put on clothes to which they have no right are certainly worthy of censure. It would really be just as suitable to dress up their coachmen and footmen in college caps and gowns as to array their maid servants as hospital nurses. Another reason why so many nurses at present are out of love with uniform is that some even of the rightful wearers do not always bear in mind that uniform conduct should go with uniform cloaks. Nothing grates on a woman of refinement more than to meet a professional sister—also in uniform—dressed untidily and behaving in a loud and vulgar way in the street. Again, there are times when it is against good taste to wear uniform. A case in point is that of a private nurse who sprang from the humbler ranks of social life and whose "young man" fell ill and was admitted to a hospital. She used to visit him on the authorised days and when he was convalescent they sat together in the grounds interspersing the conversation with frequent demonstrations of affection. This young woman was always dressed in her uniform. Our friend, the casual observer, might again be supposed to draw his own conclusions. There are some people who never can and never will see the fitness of things, but if they would only remember that uniform conduct should go with uniform cloaks, much would be done to make the wearing of uniform once more a pleasurable convenience to the sensible majority. The uniforms of members of his Majesty's navy and army are protected from insolent imitators, perhaps some day a like protection may be afforded to nurses' uniforms. The sooner the better.

### Presentations.

Ilkley Hospital and Convalescent Home. — The matron (Miss E. M. Edwards) was presented on Saturday last with a handsome silver butter-dish, with knife, by the domestic staff of the hospital. At the same time the assistant matron (Miss Laura Ulph) was presented with a beautiful silver-mounted scent-bottle. Kind wishes were expressed for a long and happy life for both. Needlework of most varied descriptions have been sent to both by patients as they have come to hear of the double resignation.

Kingston-on-Thames Union Infirmary.—Last week Miss Van Raalte, the night superintendent of the Union Infirmary, Kingston-on-Thames, who is leaving to take up private nursing, was presented with a gold chain and pendant by the staff.

Royal Halifax Infirmary.—Miss Fox on leaving the Royal Halifax Infirmary to take up private work was the recipient of various tokens of goodwill. Among the presents was an elegant silver-mounted Russia leather purse, the gift of the matron; a full-sized lady's dressing-case, from the sisters and nurses; and a leather chatelaine from her nursing staff.

Southwark Infirmary.—Dr. J. Stewart Richards, Medical Superintendent Southwark Infirmary, has been presented with a framed illuminated address and gold watch and chain by the entire staff of the Infirmary with heartiest congratulations and good wishes as a mark of regard and esteem on the occasion of his marriage.

### For Reading to the Sick.

FAITHFULNESS.

Blessed are those who die for God
And earn the Martyr's crown of light;
Yet he who lives for God may be
A greater Conqueror in His sight.

*A. Procter.*

Thy life that has been dropped aside
Into Time's stream, may stir the tide,
In rippled circles spreading wide.

The cry wrung from thy spirit's pain
May echo on some far-off plain,
And guide a wanderer home again.

Fail—yet rejoice; because no less
The failure that makes thy distress
May teach another full success.

*Ibid.*

God hath given each one of us a work to do, and what He asks for is not success—as men count success—but faithfulness. When our Lord looks down from heaven upon His Church, His message now, as of old, is not "be successful," but "be Thou faithful unto death."

So many of God's saints have seemed, for a time, to fail as men count failures.

What were the martyrdoms of earlier or of recent times but apparent failures? and yet the blood of the martyrs is the seed of the Church. Why? Because in the sight of God "success" counts for nothing, but faithfulness is everything. . . . .

Must we not, then, bring our tangled fragmentary lives here and now beneath the shadow of the Cross, and offer them up to the Crucified, and ask Him to perfect them and complete them?

Take, O Lord, the tangled threads of my life: my high aims and aspirations with my poor and miserable accomplishments, my many resolutions and their repeated collapse. Take all this tangle of the past: the years of more or less wasted opportunity. Take them; forgive all that has been wrong, perfect all that is incomplete or unworthy. Gather up the fragments that may yet remain to me of life—from henceforth let me live a life with the single desire of pleasing THEE!—*Randolph.*

What though the future with its unknown depths
Be hidden from my sight,
I know that its untrodden paths
Lead onward into light.

Yes, I will trust Thee: Thou didst once on earth
Carry our griefs alone;
Thou soughtest comforters to help,
And friends, but they were gone.

Thou knowest all my need: upon Thy care
I utterly depend;
Thy patience, that has borne the past,
Will keep me to the end.

*C. M. Noel.*

### Echoes from the Outside World.

### Movements of Royalty.

On Saturday the King of Portugal returned to Sandringham, in order to be present at the celebration of Queen Alexandra's fifty-eighth birthday on Monday. The day was one of leaden skies and drenching rain, but the rejoicings were of a particularly happy character. The customary dinner of the labourers on the estate was held, some 600 guests being invited. Later on 500 children were entertained, and the Queen herself, accompanied by King Edward, visited the happy gathering. At Windsor the bells of St. George's Chapel were rung, and the mayor sent a greeting to her Majesty "from the loyal burgesses of the borough of Windsor" Salutes were also fired in London, and flags were hoisted at the public offices and foreign Embassies. In Copenhagen the day was marked by the flying of flags and by a dinner party at the palace. At the principal seaports in England and the Colonies the ships were dressed in bunting. On Saturday morning the Duke and Duchess of Connaught left London for the East, and they are not expected to return until the spring. They first of all proceed to Egypt, where the Duke is to open the Assuan Dam, and then go on to Delhi to be present at the Coronation Durbar. A large number of friends and relatives, including their two daughters Princesses Margaret and Victoria Patricia, Princess Henry of Battenberg, and the Duke of Cambridge saw them off at Victoria. At Genoa the Duke and Duchess embarked on board the *Renown* battleship for Port Said.

### The War in Somaliland.

Preparations to advance still go on in Berbera. The place has a fine harbour, and two steamers arrive each week with men and stores. General Manning—a small dark-eyed exceedingly active man—is very busy completing final arrangements for provisioning the line of forts, but the difficulties of the Bohotle route are great. It crosses a waterless tract 120 miles wide, and where water can be obtained, the column will probably have to fight to gain possession of the wells, and to run the risk of the water being poisoned by the Mullah by means of dead camels. The Mullah in his raiding expeditions slays women and children equally with men, young and old. He has written letters to the authorities saying that he has no desire to fight the English and will make peace if we acknowledge his independence, and grant him a seaport east of Berbera, with freedom of trade. But if we want war he hopes we will send out white troops as the swords of his men have grown blunt with slaying blacks. To this he adds other insolent messages. The troops at the Mullah's disposal are said to be 30,000, but he will not probably employ more than 15,000 as his fighting strength. The little value of the Somali levies which we are endeavouring to bring into order is demonstrated daily, and rigorous punishment has to be resorted to in order to insure discipline.

### The Lady Companion of the Imperial Service Order.

The explanation of the fact that only one lady received the new Civil Order of Distinction is that no one who has not completed at least twenty-five years of meritorious service is eligible, and that a quarter of a century ago the number of women who were allowed to serve the State was almost infinitesimal. Miss M. C. Smith, the first Lady Companion of the Imperial Service Order, joined the Savings Bank Department of the General Post Office in Queen Victoria Street 30 years ago. At that time she had a small staff of girls under her; now she has nearly nine hundred. The decoration which Miss Smith is entitled to wear is a medallion of gold and enamel, bearing on one side the Imperial and Royal cipher, and on the reverse, "For Faithful Service," executed in dark-blue enamel, surrounded by a wreath of laurel surmounted by the Imperial Crown. The Companions of the Order are not to exceed 425, and it is naturally hoped that ultimately the proportion of women to men may considerably exceed that of 1 to 100.

### The Late Dr. Parker.

Following rapidly after the death of the Rev. Hugh Price Hughes, a famous Wesleyan minister in the prime of life, comes that of the Rev. Dr. Joseph Parker, a yet more celebrated Congregationalist minister, who had passed his 72nd year. Dr. Parker was the son of a stonemason, who by the exercise of abilities and application, became the pastor of the City Temple, which, indeed, owed its existence to his efforts. He came from Manchester to London in 1869, and for three years officiated at a dingy chapel in the Poultry, which, however, had historic associations. When the site was sold for £25,000 it was decided to build the well-known edifice on Holborn Viaduct at a cost of £70,000, the whole of which was paid off three years after the City Temple was finished, and since the day of its opening it has been filled with admirers of Dr. Parker from all parts of the world. Mr. Gladstone and Lord Rosebery may be mentioned among his occasional hearers.

### Royal Water Colour Society.

The Royal Water Colour Society opened on Monday. Visitors upon first entering will be struck by a certain subtle difference in the appearance of the rooms which, after a few minutes, they will realise is caused by the partial abolition of white mounts to the pictures. It used to be a rule of the society that drawings for the winter exhibition must be surrounded by white mounts. This year a free option is allowed. The most remarkable pictures are those sent by the veteran artist, Mr. Albert Goodwin. One, "Venice before the Fall of the Tower," is naturally especially interesting. It was not surprising to learn that it was sold as soon as exhibited. Another picture is "Cairo," and in a third the artist goes as far away as Port Antonio, Jamaica, to paint his "Fireflies." "A Dream Idyll," by Mr. Edward Hughes will attract considerable discussion. It represents a female figure with red-gold hair on a winged horse in a deep blue sky, passing, at night, over a sleeping city. The picture does not of necessity tell its own tale, though it instantly arrests attention, but it is an illustration of one of the fables in the "Nights of Straparola." A work by the most recently elected lady Associate, Miss Fortescue Brickdale, will be viewed with interest. It is entitled, "The Three Daughters of Time," and in its beautiful Italian frame it has been given a place of honour. Other works which should be noted are: "The Dove Cage," by Mr. Edwin Alexander; "A Cornish Sea," by Mr. Napier Hemy; "An Ebbing Tide," by Mr. Robert W. Allan; "Sleeve More, Achill," by Mr. C. B. Phillips; and "The Autumn Evening, Whitehall," by Mr. Herbert Marshall.

### A Woman's Remarkable Career.

A lady has just died at Cheltenham whose life in her younger days was a series of adventures. Mrs. Francis Isabella Duberly was the wife of an officer who was fortunate enough to obtain permission from the Government to accompany her husband through the Crimean war, and she was present at the battles of the Alma, Balaclava, Inkerman, Tchernaya and the siege of Sevastopol. She wrote a book giving details of the now well-known scandals concerning the supply of necessaries to the troops at the Crimea, which, at the time of its publication, attained some notoriety. Following her Russian experiences Mrs. Duberly followed her husband out to India, and passed through the terrible days of the Indian Mutiny. On one occasion she rode over 3,000 miles on horse-back to cantonments.

### Notes and Queries.

**The Editor is always willing to answer in this column, without any fee, all reasonable questions, as soon as possible.**

**But the following rules must be carefully observed:—**

**1. Every communication must be accompanied by the name and address of the writer.**

**2. The question must always bear upon nursing, directly or indirectly.**

**If an answer is required by letter a fee of half-a-crown must be enclosed with the note containing the inquiry, and we cannot undertake to forward letters addressed to correspondents making inquiries. It is therefore requested that our readers will not enclose either a stamp or a stamped envelope.**

*Maternity.*

(77) Will you please tell me how the following case could be called normal? The lying-in patient on the seventh day had a temperature of 102, and, being unable to lift the cup of milk from the locker, had to be fed by a feeding cup. On that day a drug was injected to cause sleep.—*A. H. F.*

It is quite impossible to answer such a questien as this. First of all was it really called normal? or was the term merely applied to the presentation?

I wrote to a lady who had asked my terms, saying that they were from £10 10s. She then informed me of the time she would require my services, and asked what my fee would be for the four weeks. I replied that it would be £12 10s., unless she were the wife of a doctor. Then she wrote saying that my fee was a little too high for her. I answered by return that, as I had no work for the date, I would accept the £10 10s. She replied that her doctor had engaged a nurse for her. Can I claim any part of the fee?—*A Distressed Nurse.*

There was no definite engagement, so you cannot claim anything. It would be better to explicitly state in the first instance that your fee is £12 10s., but that you make a reduction to the wives of medical men.

Will you kindly tell me if there are any classes near the above address where I could be coached for the L.O.S. examination?—*E. G.*

The Nurses' Club, 12 Buckingham Street, Strand, W.C., will send you syllabus of maternity lectures, and the fees for the same.

Will you kindly tell me what is the practice of midwifery which obtains in Germany? As the unqualified practitioner on whom the poorer classes in England generally rely is not permitted to practise there are appointments of accoucheurs made by Government? Will not the passing of the "Midwives Bill" necessitate some such appointment as that of parish midwife when it comes into operation?—*R. D.*

It is quite useless to speculate as to what the Government of this country will, or will not, do when the Midwives Bill comes into operation on the 1st of April. You would be able to follow the question by joining the Society for Promoting the Registration of Midwives, 12 Buckingham Street, Strand, W.C.

*Etiquette.*

(78) Will you kindly tell me if it is etiquette in private nursing to address the doctor as "doctor" or "sir?"—*E. E. B.*

It perhaps does not much matter provided you obey his instructions as regards the patient, but the proper mode of address is "Sir." Few things are more absurd than the meaningless addition of the word "Doctor" to every sentence addressed to a medical man which some people indulge in.

*Hospital Training.*

(79) I am anxious to become a nurse. Would you advise my watching advertisements or applying to hospitals? Please give me addresses of one or two London hospitals.—*L. W. E.*

See "The Nursing Profession: How and Where to Train."

I am desirous of entering one of the London hospitals to be trained as a nurse. I understand that there is a preliminary examination, and I should be glad to know what text-books would be most useful to study before being admitted as probationer to St. Bartholomew's Hospital.—*Preparation.*

If you have been accepted on probation, the matron will advise you as to the books you should study.

Will you kindly tell me of a provincial hospital which gives a three years' certificate and lectures? I have had nearly two years' training, but as I have had no lectures I find that the certificate is of little value.—*F. F.*

See "The Nursing Profession: How and Where to Train." You will then be sure that you can rely on the certificate as the conditions of training in each school are given.

*Cancer.*

(80) Will you kindly tell me if cancer is being treated anywhere in England by the X-rays, and also to whom I could apply for particulars?—*I. E. R.*

Yes, at most hospitals. Apply to the Middlesex Hospital or the Cancer Hospital, Fulham Road, S.W. Both make a special study of this disease.

*Steriliser.*

(81) I am a hairdresser, and would esteem it a favour if you would tell me of a powerful steriliser for razors, scissors, brushes, etc.—*Antiseptic.*

Heat. Any of the surgical instrument makers would be pleased to advise you on this matter.

*Floors.*

(82) Can large polished floors be kept in order without kneeling? I have tried the large weighted brushes, but they become matted, and are almost useless directly.—*Rheumatism.*

It is quite possible to keep the brushes clean. Perhaps too much wax is rubbed on to the floor in the first place. The golden rule is a little wax and much elbow grease.

*Baby Carrier.*

(83) Could any reader of The Hospital inform me where I could purchase a baby-carrier? It is made like a small hammock and is fastened on to the shoulders by strips of webbing.—*E. P.*

Can any of our readers give the required information?

*Male Nurses.*

(84) Can you tell me of a good institute where men are trained in medical nursing? I have some experience in mental nursing.—*Anxious.*

You should apply to the National Hospital for the Paralysed and Epileptic, Queen's Square, Bloomsbury, W.C., for particulars.

*Employment.*

(85) I have qualified myself in massage in several branches, and in physical culture. Will you kindly tell me how I can make a start? Ought I to join a nursing home, or start for myself by calling on medical men?—*Perplexed.*

All depends on circumstances. If you are living at some locality where the services of a masseuse are needed, write or call on the medical men; but if you will have to enter into competition with others of the same calling as yourself join a home until you gain experience.

I hold certificates for massage and electricity and wish to work in Birmingham. Would you kindly tell me the right way to get work? Should I have cards printed and send them to the doctors and chief residents in the district?—*Nurse Dorothy.*

Certainly to the doctors, and get as many private introductions as possible.

*Massage.*

(86) I should be glad if you will tell me if there is any institution where I could get training in massage free.—*E. E. W.*

The West End Hospital for Diseases of the Nervous System, 73 Welbeck Street, W., gives practical application of massage and electricity, free to nurses in return for one year's service.

*Lupus.*

(87) 1. I am a masseuse, but not a trained nurse, and I would be glad if you could kindly tell me where I could get a few lessons on the instrument for curing lupus, and if so, what is the fee? 2. I would also be glad to know if you think it would be wise to join any society in London for masseuses, and tell me what is the cost?—*R. B.*

1. It would be quite impossible to get a few lessons in the management of the Finsen Light apparatus for the cure of lupus, for that, we presume, is the instrument to which you refer. 2. You might join the Society of Trained Masseuses, 12 Buckingham Street, Strand, W.C.

**Standard Nursing Manuals.**

"The Nursing Profession: How and Where to Train." 2s. net; post free 2s. 4d.

"A Handbook for Nurses." (Illustrated). 5s.

"Nursing: Its Theory and Practice." (New Edition.) 3s. 6d.

"Nursing in Diseases of Throat, Nose, and Ear." 2s. 6d.

"Surgical Ward Work and Nursing." (Revised Edition.) 3s. 6d. net; post free, 3s. 10d.

"Art of Massage." (Second Edition.) 6s.

"Elementary Physiology for Nurses." 2s.

"Elementary Anatomy and Surgery for Nurses." 2s. 6d.

"Practical Handbook of Midwifery." 6s.

"Mental Nursing." 1s.

"Art of Feeding the Invalid." 1s. 6d.

All these are published by the Scientific Press, Ltd., and may be obtained through any bookseller or direct from the publisher, 28 and 29 Southampton Street, London, W.C.

